# Expert consensus on nutrition and lower-carbohydrate diets: An evidence- and equity-based approach to dietary guidance

**DOI:** 10.3389/fnut.2024.1376098

**Published:** 2024-02-29

**Authors:** Jeff S. Volek, William S. Yancy, Barbara A. Gower, Stephen D. Phinney, Joanne Slavin, Andrew P. Koutnik, Michelle Hurn, Jovonni Spinner, Mark Cucuzzella, Frederick M. Hecht

**Affiliations:** ^1^Department of Human Sciences, The Ohio State University, Columbus, OH, United States; ^2^Department of Medicine, Duke University, Durham, NC, United States; ^3^Department of Nutrition Sciences, University of Alabama at Birmingham, Birmingham, AL, United States; ^4^Independent Researcher, Elk Grove, CA, United States; ^5^Department of Food Science and Nutrition, University of Minnesota, St. Paul, MN, United States; ^6^Sansum Diabetes Research Institute, Santa Barbara, CA, United States; ^7^The Dietetian’s Dilemma, Portland, OR, United States; ^8^Beacon Public Health, Riverland, MD, United States; ^9^Department of Family Medicine, West Virginia University, Martinsburg, WV, United States; ^10^Osher Center for Integrative Health, University of California, San Francisco, San Francisco, CA, United States

**Keywords:** low-carbohydrate, dietary guidelines, eating patterns, high-fat, insulin resistance, obesity, type 2 diabetes, health equity

## Abstract

There is a substantial body of clinical evidence supporting the beneficial effects of lower-carbohydrate dietary patterns on multiple established risk factors associated with insulin resistance and cardiovascular diseases in adult populations. Nutrition and health researchers, clinical practitioners, and stakeholders gathered for, “The Scientific Forum on Nutrition, Wellness, and Lower-Carbohydrate Diets: An Evidence- and Equity-Based Approach to Dietary Guidance” to discuss the evidence base around lower-carbohydrate diets, health outcomes, and dietary guidance. Consensus statements were agreed upon to identify current areas of scientific agreement and spotlight gaps in research, education, and practice to help define and prioritize future pathways. Given the evidence base and considering that most American adults are living with at least one nutrition-related chronic disease, there was consensus that including a lower-carbohydrate dietary pattern as one part of the Dietary Guidelines for Americans could help promote health equity among the general population.

## 1 Introduction

The vast majority of Americans are not metabolically healthy (1). The prevalence of obesity in the United States is estimated at 41.9% (2), and obesity-related diseases are at an all-time high, with nearly 1 in 10 Americans living with type 2 diabetes (3) and nearly half of adults (48.6%) with cardiovascular disease (4). Meanwhile, nearly all Americans continue to fall short on meeting dietary recommendations for micronutrients and food groups encouraged by the Dietary Guidelines for Americans (DGA) (5), and approximately 10% of American households are classified as food insecure, defined as not having consistent, dependable access to enough food for active, healthy living (6). Traditionally marginalized population groups – racial and ethnic minority populations, tribal and indigenous communities, lower-income populations, and rural and remote populations – are disproportionately affected by diet-related chronic diseases and food insecurity (7). In 2021, 20% of Black / African American households and 16% of Hispanic / Latino households were food insecure compared to 7% of White households (8). In 2018, 14.5% of American Indian / Alaskan Natives, 12.1% of non-Hispanic Black, and 11.8% of Hispanic-Americans were living with diagnosed diabetes compared with 7.4% of non-Hispanic White Americans (9). Furthermore, mortality rates of diabetes, hypertension, heart disease, and stroke have consistently been highest among Black adults living in rural America over the past two decades, with diabetes- and hypertension-related mortality rates being 2−3 times higher than that of White adults in rural areas (10).

Cardiovascular disease (CVD) has been the leading cause of death in the United States since 1950 (11), the healthcare costs of which present an economic burden on society (12). Recent observational and experimental studies provide compelling evidence that insulin resistance is an independent risk factor for CVD, and that improvement of insulin resistance can reduce complications associated with diabetes and reduce cardiometabolic events in persons living with diabetes and among the general population (13). There is a substantial body of clinical evidence among adults supporting the beneficial effects of lower-carbohydrate dietary patterns on multiple established risk factors associated with insulin resistance and cardiovascular diseases (14). The American Diabetes Association and Diabetes Canada now recognize a low-carbohydrate eating pattern as effective for managing diabetes (15, 16).

Given the evidence base and considering that most American adults are living with at least one nutrition-related chronic disease, it seems prudent to include a lower-carbohydrate dietary pattern as one part of the DGA, alongside previously established healthy eating patterns, to help promote health equity among the general population. A barrier to including a lower-carbohydrate dietary pattern in the DGA is the current Dietary Reference Intake (DRI) for carbohydrate, which includes an Acceptable Macronutrient Distribution Range (AMDR) of 45−65% of calories (17). Scientific and physiologic rationale for the exclusion of low- or even moderate-carbohydrate diets is lacking. The inflexibility of the current DRI considered in the DGA does not align with current evidence on low-carbohydrate dietary patterns, cardiometabolic outcomes, and improved human health.

## 2 Methods

A group of expert nutrition and health researchers, practitioners, and stakeholders (Table 1) gathered on June 14, 2023 in Washington, DC for, “The Scientific Forum on Nutrition, Wellness, and Lower-Carbohydrate Diets: An Evidence- and Equity-Based Approach to Dietary Guidance” (The Forum) to discuss the evidence base around lower-carbohydrate diets, health outcomes, and dietary guidance. The goals of the forum were to: (1) catalyze a dialogue among leading nutrition and health researchers and practitioners to evaluate the evidence base around lower-carbohydrate diets and health outcomes; (2) bring together key stakeholders to identify collaboration and research opportunities to drive progress; (3) identify current areas of scientific agreement and spotlight gaps in research, education, and practice to help define and prioritize future pathways; and (4) determine key areas of nutrition security and health equity that may be addressed by a lower-carbohydrate dietary pattern. Experts presented results from peer-reviewed and published systematic reviews, randomized controlled trials, prospective cohort studies, cross-sectional analyses, and case studies. Following presentations given by experts in the field, statements pertaining to lower-carbohydrate diets were presented at the Forum by a moderator who asked all participants to vote, based on the evidence that was presented to them, with, “Agree, disagree, or agree with edits.” Voting participants included the presenters as well as trained scientists and representatives from academia, professional, and non-profit organizations. Voting was based on the totality of the evidence presented. Statements with 100% agreement were considered consensus statements. Statements with disagreement or agreement with edits were modified until 100% consensus was reached. Statements that did not gain consensus, either because of time constraints or because of disagreement, were documented.

**TABLE 1:** Speakers and participants from the Scientific Forum on Nutrition, Wellness, and Lower-Carbohydrate Diets: An Evidence- and Equity-Based Approach to Dietary Guidance.

Name	Affiliation	Role at forum
Jonathan Bates	The Nutrition Coalition	Voting participant
Mark Cucuzzella, MD	West Virginia University	Voting participant
Barbara A. Gower, PhD	University of Alabama at Birmingham	Steering committee member, speaker, and voting participant
Elizabeth Hanna, MS, RDN, CDCES	American Diabetes Association	Voting participant
Frederick M. Hecht, MD	University of California, San Francisco	Voting participant
Michelle Hurn, RD	The Dietitians Dilemma	Speaker and voting participant
Andrew P. Koutnik, PhD	Sansum Diabetes Research Institute	Voting participant^1^
Vyvyane Loh, MD	Transform Alliance for Health	Speaker and voting participant
Stephen D. Phinney, MD, PhD	Virta Health, Professor emeritus, University of California	Speaker and voting participant
Joanne Slavin, PhD, RD	University of Minnesota	Voting participant^1^
Jovonni Spinner, PhD	Beacon Public Health	Speaker and voting participant
Jeff S. Volek, PhD, RD	The Ohio State University	Steering committee member, speaker, and voting participant
Taylor Wallace, PhD	Think Healthy Group, George Mason University	Speaker, non-voting participant^2^
William S. Yancy, Jr., MD, MHS	Duke University	Steering committee member, speaker, and voting participant

^1^Drs. Slavin and Koutnik viewed the presentations asynchronously and participated in virtual voting for consensus statements;

^2^Dr. Wallace presented a scoping review at The Scientific Forum on Nutrition, Wellness, and Lower Carbohydrate Diets and is not included in the consensus statements.

## 3 Defining lower-carbohydrate dietary patterns

There currently is no standard definition for a “low-carbohydrate” diet that has been universally accepted among the scientific community (14). The AMDR for carbohydrate is 45−65% of daily caloric intake (17), with a Recommended Dietary Allowance (RDA) of 130 g of carbohydrate per day across all age and sex groups. The basis for this amount of dietary carbohydrate was cited as being due to the average amount of glucose utilized daily by the brain (18). Evidence demonstrates, however, that the brain can utilize other substrates such as ketones, and glucose can be derived from non-carbohydrate sources via gluconeogenesis, to adequately sustain energy needs regardless of carbohydrate intake (19). The lack of a universally accepted, standard definition for a low-carbohydrate diet creates a barrier for determining dietary guidance regarding low-carbohydrate dietary patterns. For example, without a standard definition to dictate low versus high carbohydrate diets, the 2020−2025 Dietary Guidelines Advisory Committee (DGAC) instead focused on whether, and the extent to which, the proportion of macronutrients below or above the AMDR impacted a variety of health outcomes (20). The 2020−2025 DGAC concluded there was insufficient evidence available to determine the relationship between non-energy-restricted diets based solely on macronutrient distribution proportions outside of the AMDR for at least one macronutrient and risk of most health outcomes assessed (20).

Based on the available literature, the attendees of the Forum agreed that lower-carbohydrate dietary patterns included those that contained less than 130 g per day [(14), Table 2]. This was in alignment with a scientific statement published by the National Lipid Association Nutrition and Lifestyle Task Force on the effects of low- and very-low-carbohydrate diets for the management of body weight and other cardiometabolic risk factors (21). Attendees of the Forum agreed that future research should utilize a standard definition of lower-carbohydrate diets in terms of grams from carbohydrates, include measurement of respiratory quotient or ketones to assess adherence, and utilize biomarkers to determine the level of carbohydrate restriction necessary to induce metabolic benefits that are stratified by individual needs and health status (Table 3).

**TABLE 2:** Consensus definitions and categories of lower-carbohydrate dietary patterns proposed by attendees of the Scientific Forum on Nutrition, Wellness, and Lower-Carbohydrate Diets: An Evidence- and Equity-Based Approach to Dietary Guidance.^1^

Diet description	Energy	Carbohydrate (g/d)
**Lower-carbohydrate dietary patterns**		
Very-low carbohydrate, high-fat ketogenic diet	<10%	20−50
Low-carbohydrate diet	10−25%	50−129
**Higher-carbohydrate dietary patterns**		
Moderate-carbohydrate diet	26−44%	130−224
High-carbohydrate diet	45−64%	225−324
Very-high carbohydrate diet	≥65%	≥325

^1^Based on a 2,000 kcal/day eucaloric diet.

**TABLE 3:** Consensus statements regarding nutrition considerations for lower-carbohydrate diets.

Statements
Future research should utilize a standard definition of lower-carbohydrate diets in terms of grams from carbohydrates and parallel biomarkers to determine the level of carbohydrate restriction necessary to induce metabolic benefits stratified by individual needs and health status.
Macronutrient quality with a focus on nutrient-dense foods is an important consideration within the context of a low- or very-low carbohydrate diet.
Vegetables, fruits, legumes / beans, and whole-grains can be valuable sources of nutrient-dense carbohydrates.
Protein and fat sources can include meat / poultry / seafood, whole eggs, tofu / combined plant proteins, nuts / seeds, dairy foods, non-dairy alternatives, and cold-pressed oils / fats.

## 4 State-of-the-science on benefits, risks, and gaps in research pertaining to lower-carbohydrate dietary patterns

Lower-carbohydrate dietary patterns of less than 130 g per day of carbohydrate have been demonstrated to result in more favorable effects on average weight loss and certain risk markers for cardiovascular disease when compared with low-fat dietary patterns (22, 23). A systematic review and meta-analysis of 38 clinical trials that assessed 6,499 adults indicated that lower-carbohydrate dietary patterns were effective at improving weight loss, HDL-cholesterol, and triglycerides in adults at 6−12 months when compared with low-fat diets, with low-fat diets favoring reductions in total- and LDL-cholesterol compared with lower-carbohydrate diets (22). A systematic review and meta-analysis of longer-term randomized controlled trials conducted from 8 weeks to 24 months in people living with overweight and obesity demonstrated that lower-carbohydrate dietary patterns resulted in significantly greater improvements in weight loss and reductions in predicted atherosclerotic cardiovascular disease events as determined by pooled cohort equations developed by the National Heart, Lung, and Blood Institute (23). Another meta-analysis of 25 clinical trials conducted in people with BMI greater than 30 kg/m^2^ indicated that lower-carbohydrate diets were associated with greater short-term weight loss than non-carbohydrate restricted diets (24). Furthermore, whereas no significant differences were detected in total- or LDL-cholesterol or blood pressure, significant increases in HDL-cholesterol and reductions in triglycerides were observed at 18−30 months (24). One randomized, parallel-group trial that compared a very-low carbohydrate (<40 g/d) and low-fat diet (<30% fat; <7% saturated fat) found that the estimated 10-year Framingham risk score had significant decreases in the very-low carbohydrate group, but not the low-fat group at 6 and 12 months (25). The risk score utilizes a holistic approach to calculating coronary heart disease (CHD) risk by accounting for factors such as total and HDL-cholesterol and systolic blood pressure, rather than relying on a single biomarker, such as LDL-cholesterol, to determine risk. The results of these studies indicate lower-carbohydrate dietary patterns result in significant reductions in weight and improvements in risk markers for cardiovascular disease (Table 4).

**TABLE 4:** Consensus statements regarding the state-of-the-science on benefits, risks, and gaps in research pertaining to lower-carbohydrate diets.

Statements
Multiple clinical trials in humans consistently demonstrate that low-carbohydrate diets can result in significant improvements in insulin resistance and other risk markers for cardiovascular disease.
Low-carbohydrate dietary patterns are supported by a large number of well-designed research studies funded by a variety of sources (federal entities, foundations, industry, university, etc.) and published across a wide range of independent, peer-reviewed journals.
Well-formulated low-carbohydrate eating patterns are consistent with normal human physiology and comparable to other dietary patterns for body composition outcomes such as percent body fat, ectopic / unhealthy fat, and lean body mass.
Current evidence indicates well-formulated low-carbohydrate diets are comparable in safety and efficacy to other dietary patterns regarding areas of concern (e.g., dyslipidemia, micronutrient deficiencies, adverse effects on kidneys or bone health).
Low-carbohydrate diets are safe for the general population but may require knowledgeable medical supervision for a set period of time for certain conditions (e.g., type 1 diabetes, people with type 2 diabetes on insulin or glucose lowering medications or sulfonylureas, etc.).
Low-carbohydrate diets are helpful for addressing risk factors for cardiovascular disease and are not associated with significant adverse effects.

In a systematic review and meta-analysis of eight randomized controlled trials, a very-low carbohydrate diet was demonstrated to beneficially affect weight loss, waist circumference, glycated hemoglobin, triglycerides, and HDL-cholesterol in patients with type 2 diabetes (26). A randomized controlled trial conducted in 36 patients with metabolic syndrome demonstrated that even without instructions to reduce calories, participants on a very-low carbohydrate diet or a low-fat diet reduced caloric intake and lost weight, with those enrolled in the very-low carbohydrate diet group experiencing significantly more weight loss than those enrolled in the low-fat diet group after 12 weeks (27). The very-low carbohydrate diet also resulted in more significant improvements in abdominal fat and serum triglycerides, HDL-cholesterol, triglyceride-to-HDL ratio, apoB, ApoB/ApoA-1, small LDL-cholesterol, glucose, HOMA-IR, leptin, multiple pro-inflammatory cytokines, and total saturated fatty acids, despite dietary intake of saturated fat being three-times higher than the low-fat diet (27). In a three-way cross-over controlled-feeding trial conducted in 16 adults living with obesity and metabolic syndrome, a low carbohydrate diet resulted in enhanced fat oxidation and significant improvements in serum triglycerides, HDL-C, and small LDL-cholesterol, independent of weight loss, when compared with eucaloric moderate or high-carbohydrate / high-fat diets after 4 weeks (28). In a highly controlled short-term trial conducted in 10 patients living with obesity and type 2 diabetes, an *ad libitum* very-low carbohydrate diet resulted in significant reductions in fasting blood glucose, HbA1c, and increased insulin sensitivity (∼75%) after 14 days (29). The results of these trials indicate that metabolic syndrome and type 2 diabetes may be reversed by following a very-low-carbohydrate dietary pattern, with or without significant weight loss.

A ketogenic diet has repeatedly been shown to decrease indicators of inflammation. Individuals with type 2 diabetes prescribed a ketogenic diet demonstrated lower serum c-reactive protein and white blood cell count (30), as well as significant reductions in 15 out of 16 inflammatory/immune modulators measured after 1 and 2 years (31). This anti-inflammatory effect is consistent with earlier findings that showed 7 out of 14 inflammation/immune modulators were reduced to a greater degree with a ketogenic diet compared to an energy-restricted low-fat diet after 12 weeks (27).

Despite robust clinical evidence indicating beneficial effects of well-constructed low-carbohydrate diets on markers of metabolic health, the long-term safety of consuming low-carbohydrate diets remains controversial. A systematic review and meta-analysis of observational studies conducted with at least 1 year of follow-up determined low-carbohydrate diets were associated with significantly higher risk of all-cause mortality, and not significantly associated with risk of cardiovascular mortality and incidence (32). Researchers reported that results were tentative, based on data with high heterogeneity and publication bias, and thus had to be interpreted with caution (32). These results highlight a gap in long-term prospective research on the safety and efficacy of low-carbohydrate diets. Results from a more recent systematic review and meta-analysis of randomized clinical trials, however, found that in adults with type 2 diabetes, patients adhering to a low-carbohydrate diet may experience remission of diabetes without adverse consequences (33). More research from an adult population free from disease would add tremendous value to the existing literature.

## 5 Tailoring the dietary guidelines to current nutrition concerns

Nutrition security, consistent and equitable access to safe and affordable foods that promote well-being, prevent disease, and if needed, treat disease, particularly among traditionally marginalized population groups, is a national priority (34). One strategy to ensuring nutrition security and health equity among Americans is by providing guidance on more tailored approaches that address the most prevalent diet-related diseases (35). The 2025−2030 DGAC is examining all evidence through a “health equity lens” to provide guidance that supports nutrition security and meets the needs of all Americans (34).

A diet modeling study designed to evaluate the effect of targeted food substitutions that mimicked popular diets on overall diet quality as measured by the Healthy Eating Index (HEI)-2015, determined that carbohydrate restricted diets were associated with poor diet quality based on dietary data from 34,411 adults acquired from the NHANES, 2005−2018 (36). When the authors modeled replacement of foods highest in added sugar, sodium, saturated fat, and refined grains with alternative foods, however, there was an increase in diet quality and decrease in energy intake for most of the dietary patterns that were evaluated, with no significant difference between the modeled HEI-15 for restricted-carbohydrate diets and the diet of the general population (36). Results from a series of systematic reviews and meta-analyses of prospective cohort studies that aimed to establish an evidence base for quantitative recommendations for intakes of dietary fiber demonstrated that implementation of recommendations to increase dietary fiber and replace refined grains with whole grains could reasonably be expected to benefit human health outcomes (37). A recent prospective cohort study that utilized data from the Nurses’ Health Study, Nurses’ Health Study II, and Health Professionals Follow-Up Study observed that whereas consumption of refined grains and starchy vegetables was associated with weight gain, carbohydrate intake from whole grains, fruit, and non-starchy vegetables was not (38). The results of these studies highlight the need for well-constructed lower-carbohydrate dietary patterns that can help close gaps in micronutrient and fiber intake that exist in the general population, which can be achieved by incorporating a wide variety of nutrient dense whole foods while minimizing the consumption of refined carbohydrates.

A proposed lower-carbohydrate dietary pattern published in 2023 that maintained the 1,800 kcal per day level of the Healthy U.S. Dietary Pattern proposed by the DGA and provided adequate fiber, potassium, calcium, and vitamin D showed that well-constructed lower-carbohydrate dietary patterns can be adequate and comparable in diet quality to existing DGA menu models (Table 5; 39). The proposed pattern lowered carbohydrates from 289 to 126 g by decreasing from six to one ounce equivalent per day from grains and three to one half cup equivalent per day from dairy; an increase from two-and-a-half to five cup equivalents per day from vegetables, five-and-a-half to 12-ounce equivalents per day from protein foods, and 24 to 42 g per day from oils (Table 6; 39). The proposed lower-carbohydrate dietary pattern fits within the RDA for carbohydrate and the USDA Thrifty Food Plan, which is the estimated cost of groceries needed to provide a healthy, budget-conscious diet for a family of four (39).

**TABLE 5:** Consensus statements regarding tailoring the Dietary Guidelines for Americans to current nutrition concerns.

Statements
Well-constructed low-carbohydrate dietary patterns (or menu models) can be adequate and comparable in diet quality to existing DGA menu models.
Adding a low-carbohydrate dietary pattern to the DGA is consistent with improved nutrition security and health equity.
Low-carbohydrate diets are helpful for addressing insulin resistance within the general population and in those with or at risk of certain metabolic impairments and diet-related diseases (e.g., cardiovascular disease, obesity, metabolic syndrome, prediabetes, type 2 diabetes, polycystic ovary syndrome, non-alcoholic fatty liver disease, neurodegenerative disorders, certain cancers, etc.).

**TABLE 6:** Proposed lower-carbohydrate dietary pattern compared with healthy US dietary pattern with daily amounts from food groups and components at 1,800 kcal/d.^1^

	Proposed lower-carbohydrate dietary pattern	Healthy US dietary pattern
**Food group or subgroup**	**Daily amount of food from each group**	
Vegetables (cup eq/d)	5	2.5
Fruits (cup eq/d)	0.5	1.5
Grains (ounce eq/d)	1	6
Dairy (cup eq/d)	0.5	3
Protein foods (ounce eq/d)	12	5.5
Oils (g/d)	42	24
Total carbohydrate (g/d)	126	289

^1^Thrifty Food Plan: in October 2021, $58.32/week for female 20-50 years old in 1-person household. In November 2021, cost for 1-day menu $6.95 using state of Colorado pricing.

A descriptive study utilizing data from average Australian adults aimed at assessing the nutrient intake of a lower-carbohydrate dietary pattern of less than 130 g of carbohydrate daily with 15−25% of total energy from protein and the remaining from fat demonstrated that lower-carbohydrate meal plans exceeded Australian / New Zealand nutrient reference value (NRV) thresholds for micronutrients with exception to iron for females, which reached 86−98% of the threshold (40). Saturated fat exceeded the 10% NRV by 0.6% in the meal plan formulated for males (40). In another study aimed at presenting practical meal plans for meeting the NRV for 27 essential vitamins, minerals, and fatty acids using a lower-carbohydrate dietary pattern derived from the leading low-carbohydrate food sources in the Food Standards Australia New Zealand, Australian Food Composition Database, a well-constructed lower-carbohydrate meal plan was shown to meet or exceed the highest NRVs by greater than or equal to 90% for the vast majority of nutrients that were measured (41). These studies showed that well-constructed lower-carbohydrate dietary patterns can supply sufficient micronutrients to the diets of most adults. A wide-range of nutrient-rich whole foods including meat, poultry, seafood, whole eggs, tofu/plant proteins, nuts/seeds, dairy foods, non-dairy alternatives, and oils/fats can fit into a low-carbohydrate dietary pattern.

We acknowledge that within the parameters of a low-carbohydrate dietary pattern (Table 2), there is a wide range of food choice that could be selected by individuals ranging from nutrient poor to nutrient dense. As with any eating pattern, educational efforts should prioritize selecting higher quality food items that are nutrient dense.

## 6 Addressing barriers, concerns, and compliance with lower carbohydrate dietary patterns

Barriers to acceptance of lower-carbohydrate dietary patterns include concerns related to metabolic disease associated with increased fat consumption; kidney disease; symptomatic side effects; bone loss; cancer; increased food costs; and access to foods. Systematic reviews investigating the impact of saturated fat consumption on mortality, major cancer, and cardiovascular disease outcomes indicate minute absolute changes in risk with low and very low certainty of evidence (42). Whereas saturated fat consumption has been demonstrated to raise LDL-cholesterol, it is typically larger LDL particles that are less associated with risk for cardiovascular disease (43). A one-year randomized, parallel-group clinical trial demonstrated that when compared with a low-fat diet intervention, a low-carbohydrate diet intervention resulted in greater reduction in cardiovascular risk factors in black and white adults with obesity (25). Current evidence indicates there is insufficient and inconsistent evidence to support reduction of saturated fat without consideration of the total macronutrient distribution of dietary patterns and the food matrices that deliver them (44).

Well-constructed low-carbohydrate diets should not contain excessive protein. In the two-year Dietary Intervention Randomized Controlled Trial (DIRECT) improvements to estimated glomerular filtration rate were demonstrated with similar magnitude in participants following either a low-fat, Mediterranean, or low-carbohydrate diet demonstrating that a well-constructed low-carbohydrate diet can be as safe and effective as a low-fat or Mediterranean diet at preserving or improving kidney function among people with obesity with or without type 2 diabetes (45). Researchers concluded improvements to kidney function were likely mediated by weight-loss (45). A clinical trial demonstrated that a low-carbohydrate diet did not negatively impact kidney function in women with overweight or obesity (46; Table 7).

**TABLE 7:** Consensus statement regarding addressing barriers, concerns, and compliance with lower-carbohydrate diets.

Statement
Long-term adherence with low-carbohydrate diets may be achievable and comparable to that of other healthy dietary patterns, given adequate education, resources, and support.

Hypothetical detrimental effects of low-carbohydrate dietary patterns associated with bone health and cancer risk have not been demonstrated. A systematic review of published studies evaluating the relationship between low-carbohydrate diets and bone health determined that to-date, there have not been adequately powered clinical trials conducted in humans to demonstrate either a detriment or benefit of low-carbohydrate diets on bone health (47). Rigorously controlled studies and long-term diet implementation and follow-up of individuals are necessary to adequately assess such concerns.

The cost of lower-carbohydrate dietary patterns varies widely depending on food preference/selection, but may be higher than that of other dietary patterns (48), and presents a potential barrier to adoption of lower-carbohydrate dietary patterns, particularly when considering a health-equity lens. However, a well-constructed lower-carbohydrate dietary pattern can fit into the USDA Thrifty Food Plan (39), which is described as being designed to represent the cost of a nutritious, practical, and cost-effective diet. More detailed analyses are needed to assess affordability and accessibility for various socioeconomic groups.

Despite evidence that carbohydrate restriction can significantly improve metabolic health independent of weight loss (28), concerns regarding the ability of persons to sustain a lower-carbohydrate lifestyle persist. For perspective, current adherence of the U.S. population to the current DGA, as measured by the average total HEI-2015 scores is 59 out of 100 (5). Historically, native hunting, fishing, and herding cultures were able to survive for millennia with sparse carbohydrate consumption (49–51) and a very-low carbohydrate ketogenic diet has been implemented in the successful treatment of epilepsy (52) for over a century. A recent review that aimed to evaluate the effects and tolerability of a low-carbohydrate dietary treatment for glucose transporter type 1 deficiency syndrome determined side-effects of the diet were minimal and the compliance rate was 88% at four-and-a-half years of follow-up (53). In May 2021, the Scientific Advisory Committee on Nutrition (SACN) published a risk assessment on lower carbohydrate diets for adults living with type 2 diabetes with the aim of reviewing the evidence on low-carbohydrate diets compared with UK government advice on carbohydrate intake for adults with type 2 diabetes (54). Following systematic review of the lower-carbohydrate / higher fat and / or higher protein diet scientific literature, SACN published recommendations stating a lower carbohydrate diet can be recommended by clinicians as an effective short-term option for up to 6 months for improving glycemic control and serum triglyceride concentrations in adults living with type 2 diabetes and overweight or obesity (54). The SACN further recommended that when choosing a lower carbohydrate diet, individuals living with type 2 diabetes and overweight or obesity should include whole-grain or higher fiber foods, a variety of fruits and vegetables and limit intakes of saturated fats, reflecting current dietary guidance for the general healthy adult population (54). The recommendations highlight the necessity for low-carbohydrate diets to be well-planned and consider not just the quantity, but also the quality, of the carbohydrates recommended (55). Similarly to dietary guidance in the US, the SACN recommendations also made clear that dietary guidance is intended for the general population and not written for individuals living with chronic disease, but highlighted that since the majority of individuals living with type 2 diabetes have overweight or obesity, that weight management is a primary goal for improving glycemic control and reducing the risk for cardiovascular disease. This prompted SACN to recommend that health professionals support any evidence-based dietary approach that helps individuals with type 2 diabetes to achieve long-term weight reduction (54). The SACN stated that limitations to recommending low-carbohydrate diets included a limited evidence base because of the lack of a formalized definition for low-carbohydrate diets; uncertainties in available data; and a high risk of bias in available randomized clinical trials that were included in the meta-analysis (54). Further, SACN identified gaps in knowledge that still exist, such as how the effects of lower-carbohydrate diets change depending on the quality of carbohydrate consumed, the potential impact of increasing the proportions of other nutrients on clinical outcomes related to type 2 diabetes, and effects in individuals of different ethnicities or the general population that is not living with type 2 diabetes, overweight or obesity, and the effects beyond 12-months (54).

Non-randomized controlled clinical trials that assessed the effectiveness and safety of a remote continuous care model for managing type-2 diabetes with very-low carbohydrate nutrition demonstrated that 75% of participants sustained the diet for up to 2 years (56). The very-low carbohydrate diet safely improved HbA1c, weight, systolic and diastolic blood pressures, and serum triglycerides, HDL-cholesterol, white blood cells, C-reactive protein and alanine transaminase compared with baseline with no serious adverse events reported (56). The very-low carbohydrate diet also resulted in reductions in type-2 diabetes medication (56). These effects were not detected in patients enrolled in usual care (56). Spine bone mineral density in the very-low carbohydrate group was unchanged (56). The use of glycemic control medication (with exception to metformin) was significantly reduced (56) and resolution of diabetes (reversal, 53.5%; remission, 17.6%) occurred in the continuous care group at 2 years, but not the usual care group (56). This was an important finding, particularly because the aforementioned recommendations from the SACN included a recommendation that stated adults living with T2D and overweight or obesity who change to a lower carbohydrate diet and taking diabetes medication may be at risk of hypoglycemia and to solicit advice and support from their health care team to manage that risk and make adjustments to their medication as required (54). A cross-sectional study from Sweden that aimed to assess how low-carbohydrate diets are composed in free-living adults found that low-carbohydrate dietary patterns can be sustained over time without apparent risk of deficiencies (57). The results of these systematic reviews and meta-analyses, clinical trials, and observational research demonstrate the safety and efficacy of low-carbohydrate diets that are carefully constructed and monitored with continuous care. More research investment in the effects of following lower-carbohydrate diets for longer periods of time among the general population would add value to this body of literature. Based on the current available literature, long-term adherence with low-carbohydrate diets may be achievable and comparable to that of other healthy dietary patterns, given adequate education, resources, and support.

## 7 Nutritional approaches to address metabolic health and cultural diversity of the U.S. population

Healthy People 2030, the U.S. Department of Health and Human Services Office of Disease Prevention and Health Promotion’s data-driven national objectives to improve health and well-being over the next decade, defines health disparity as “a particular type of health difference that is closely linked with social, economic, and / or environmental disadvantage.” Health disparities adversely affect groups of people who have systematically experienced greater obstacles to health based on their racial or ethnic group; religion; socioeconomic status; gender; age; mental health; cognitive, sensory, or physical disability; sexual orientation or gender identity; geographic location; or other characteristics historically linked to discrimination or exclusion (58). Leading causes of death in the US, such as CVD and cancer, are associated with diet-related risk factors that disproportionately affect racial and ethnic minorities in America (1).

Ancestry is associated with variation in genes that affect metabolism, manifest as phenotypic characters, and contribute to racial and ethnic differences in risk for chronic disease. Examples of phenotypic traits that differ with ancestry include the greater acute insulin response, greater beta-cell response, and lower hepatic insulin extraction, all of which have been well documented in Black compared to White children and adults (59, 60). In a clinical trial that aimed to determine if moderate carbohydrate restriction was beneficial for body composition and metabolic health among men and women with overweight or obesity, Black participants on the lower-carbohydrate diet (43, 18, and 39% of energy from carbohydrate, protein, and fat, respectively) for 16 weeks lost more body fat than those on the low-fat diet (55, 18, and 27% of energy from carbohydrate, protein, and fat, respectively) (60). There were no differences in outcomes in White participants following either of the two diets (60). The results of these studies indicated diet composition affected weight gain and weight loss in Black, but not White adults.

In another clinical trial designed to test whether insulin secretion affected weight loss with lower-carbohydrate and low-fat diets, change in body weight and adiposity did not differ between diet groups overall, but insulin concentration at 30 min post oral glucose challenge was an effect modifier (61). The low-carbohydrate diet produced a greater decrease in weight and adiposity than the low-fat diet after eighteen months in participants with insulin concentrations above the median at 30 min (61). This study demonstrated that variability in effects from different diets may be attributable to differences in hormonal responses. In a clinical trial aimed to determine if proinsulin secretion would be higher and associated with indices of beta-cell function in Black adults relative to White adults without type 2 diabetes, Black participants had higher indicators of beta-cell dysfunction, highlighting that Black adults may be predisposed to diabetes compared with White adults (62). Thus, offering dietary patterns with different glycemic and insulinemic profiles as part of the DGA may offer alternative opportunities to address personalized and precision nutrition for specific populations.

Type 2 diabetes is a heterogeneous disease for which there are several phenotypes that can help inform patient management (63). Genetic risk for insulin resistance may be higher in minority populations, which may contribute, in part, to higher risk for obesity and diabetes in Black (62) and Hispanic (64) populations. Lower-carbohydrate dietary patterns may be an effective means by which to lower risk and / or improve management of type 2 diabetes within these population groups.

The 2022 White House National Strategy on Hunger, Nutrition, and Health stated that the DGAC will apply a health equity lens to ensure that the 2025−2030 DGA is inclusive of people from diverse racial, ethnic, socioeconomic, and cultural backgrounds (34). With evidence indicating that lower-carbohydrate dietary patterns are associated with beneficial effects on weight maintenance, insulin sensitivity, and markers for CVD – metabolic diseases that disproportionately affect people from historically marginalized backgrounds – the inclusion of a lower-carbohydrate dietary pattern in the DGA could enhance health equity in the US (39; Table 8). In a systematic review of dietary interventions for T2D in South Asian populations, researchers concluded lower carbohydrate diets could support approaches for the treatment, but more emphasis on providing culturally relevant nutrition therapy is necessary (65). Of importance will be the inclusion of traditional and culturally relevant meal plans developed in partnership with community leaders and organizations that represent culturally diverse population groups.

**TABLE 8:** Consensus statements pertaining to nutritional approaches to address metabolic health and cultural diversity in the US population.

Statements
Current eating patterns in the DGA do not reflect an adequate range of macronutrient distribution that could benefit metabolically vulnerable sub-populations such as Black and Hispanic populations that are at greater risk for impaired glucose / insulin dynamics.
There is substantial evidence that allowing macronutrient flexibility, including a lower-carbohydrate dietary pattern, within the DGA could help address health disparities and advance health equity by providing culturally tailored dietary options that address common metabolic issues in historically marginalized communities.
Low-carbohydrate diets can be adapted to diverse ways of eating including plant-based diets and culturally relevant foodways.

## 8 Discussion

The US is comprised of a metabolically unhealthy population (1) that is falling short of meeting current dietary recommendations (5) with traditionally marginalized groups being disproportionately affected by diet-related chronic disease and food insecurity (7). Experts agree that healthy dietary patterns and regular physical activity are fundamentally crucial for maintaining good health (34). Until now, low-carbohydrate diets were seen as treatment options for people living with epilepsy or T2D and overweight or obesity (14). This statement is the first to our knowledge to synthesize the state-of-the-science on lower-carbohydrate dietary patterns using an evidence and equity approach to inform dietary guidance. The Forum agreed with statements on definitions for lower-carbohydrate dietary patterns; described their utility in achieving a healthy weight, improving risk markers for cardiometabolic diseases, and managing metabolic syndrome and T2D; addressed barriers to adoption; and highlighted the need for more tailored dietary guidance that will promote health equity among a culturally diverse population that is increasingly food insecure and living with at least one chronic condition (39).

The safety of alternative and low-calorie sweeteners in foods has been extensively studied and evaluated by regulatory bodies, globally (66). Nonetheless, concerns regarding the safety of their use persist. The use of alternative and low-calorie sweeteners in low-carbohydrate dietary patterns was not discussed at the Forum, and thus no consensus statements specific to their safety and use were developed. An expert consensus on low-calorie sweeteners published in 2020 indicated that the safety of low-calorie sweeteners as currently consumed is demonstrated by a substantial body of evidence reviewed by regulatory experts and that even higher use falls within established safety margins (66). The panel also concluded that available evidence did not support concerns regarding adverse effects of low-calorie sweeteners on sweet preference, appetite, or glucose control; to the contrary they may improve glycemic control and dietary compliance, and that data regarding the effects on gut microbiota at doses relevant to human use were limited (66).

The long-term effects of following a well-constructed low-carbohydrate dietary pattern of up to 130 g of high-quality carbohydrate per day have not been well documented in large prospective cohorts of metabolically healthy populations. This presents a limitation to our understanding and an opportunity for future research, which can also consider the socioeconomic impacts of well-constructed low-carbohydrate diets on culturally diverse populations. Based on the available literature the strengths of including a well-constructed low-carbohydrate dietary pattern as one part of the DGA include providing a dietary pattern that can promote health equity and be nutritionally adequate when compared to other recognized dietary patterns, and has been demonstrated to help achieve a healthy weight and improve risk markers for cardiometabolic diseases among culturally diverse populations.

## 9 Conclusion

Dietary guidance in America could provide more flexibility and include a wider range of carbohydrate intakes that are compatible with good health. Specifically, the DGA could include a lower-carbohydrate dietary pattern that is culturally relevant and promotes health equity among the general population in addition to existing healthy dietary patterns. Data indicate that a well-constructed low-carbohydrate dietary pattern could have positive impacts on decreasing the high prevalence of obesity, prediabetes, metabolic syndrome, and T2D while promoting health equity and food security and thus be considered as an accepted eating pattern in the DGA.

## Author contributions

JV: Writing – original draft, Writing – review & editing. WY: Writing – original draft, Writing – review & editing. BG: Writing – original draft, Writing – review & editing. SP: Writing – original draft, Writing – review & editing. JSl: Writing – original draft, Writing – review & editing. AK: Writing – original draft, Writing – review & editing. MH: Writing – original draft, Writing – review & editing. JSp: Writing – original draft, Writing – review & editing. MC: Writing – original draft, Writing – review & editing. FH: Writing – original draft, Writing – review & editing.
